# Full root-zone nitrogen fertilizer application during rice transplanting promoted root development to increase rice yield and improve nitrogen use efficiency in South China

**DOI:** 10.3389/fpls.2025.1715187

**Published:** 2026-01-19

**Authors:** Kaixin Zhang, Dengbin Fu, Zhiwei Zeng, Ying Chen, Xingna Jia, Zhenyu Tang

**Affiliations:** 1 Hubei Agricultural Machinery Engineering Research and Design Institute, Hubei University of Technology, Wuhan, China; 2 Hubei Agricultural Machinery Engineering Research and Design Institute, Wuhan, China; 3 College of Engineering, South China Agricultural University, Guangzhou, Guangdong, China; 4 Department of Agricultural Engineering Technology, University of Wisconsin-River Falls, River Falls, WI, United States; 5 Department of Biosystems Engineering, University of Manitoba, Winnipeg, MB, Canada; 6 Zhaoqing Academy of Agricultural and Forestry Sciences, Guangdong, China

**Keywords:** fertilizer application, nitrogen use efficiency, rice crop, root-zone, yield

## Abstract

**Introduction:**

The current nitrogen fertilizer application methods during rice (*Oryza sativa L*.) transplanting have many problems. To address the problems, a new method, the full root-zone fertilizer application method (RF), was applied during transplanting.

**Methods:**

To investigate the impact of the RF method compared with other fertilizer methods on rice productivity, three repeated experiments were carried in different sites in South China from 2022 to 2023, involving four treatments being four fertilizer application methods: no nitrogen fertilizer applied throughout whole growth cycle (C), RF, deep-side fertilizer application (DF), and manually broadcasting fertilizer application (BF). All treatments applied 100 kg ha^-1^ N during transplanting except C.

**Results and Discussion:**

The experiment results demonstrated that at the tillering stage, the RF method significantly enhanced rice root characteristics over the BF method, with increases of 24.41% in average root length, 34.93% in diameter, and 25.22% in total root surface area. The tiller number and the antioxidant enzymes activity under the RF method were significantly higher than the DF and BF methods during the rice growth period. The rice yield under RF method increased 13.87% to 33.41% compared with BF and DF methods. This increase was associated with superior performance in effective panicle number and seed setting rate at maturity. The RF method also significantly enhanced nitrogen use efficiency (NUE) compared to other methods, by reducing nitrogen loss and improving plant nitrogen uptake. Additionally, the RF method increased net income by 31.77% to 45.33% over the DF and BF methods by raising the total rice value.

## 1 Introduction

Rice (*Oryza sativa L*.) is vital for global food security (Mohapatra and Sahu, 2022). Its production relies on fertilizer, and nitrogen is essential. Moderate nitrogen application boosts both yield and quality (Sui et al., 2013). Traditional nitrogen fertilizer application practices typically involve broadcasting fertilizer manually before transplanting or sowing (Zhu et al., 2021). However, this method often leads to un-even nitrogen fertilizer distribution, which can result in either excessively low or high local fertilizer concentrations that inhibit rice growth (Wang et al., 2022), ultimately causing yield re-ductions. In addition, broadcasting nitrogen fertilizers on the soil surface results in significant losses during the dissolution process due to volatilization and runoff, thereby reducing NUE (Liu et al., 2020; Chen et al., 2021). Also, this approach is associated with various environ-mental issues, including air contamination (Ghaly and Ramakrishnan, 2015), soil compaction, salinization (Ondrasek et al., 2011), and water eutrophication (Chien et al., 2009). Currently, several new nitrogen fertilizer application methods are gradually replacing traditional manual broadcasting, including rotary tillage nitrogen fertilizer method, layered nitrogen fertilizer method (Zeng et al., 2020), and side-deep banding nitrogen fertilizer methods (Zhang et al., 2021).

Rotary tillage fertilizer method involves spreading fertilizer on the soil surface before rice transplanting or sowing, followed by mixing the fertilizer into the soil using a rotary cultivator (Wang et al., 2022). In recent years, some researchers have the fertilizer spreading process in this method to reduce the problem of uneven fertilizer distribution (Gyldengren et al., 2020). However, this method still faces challenges with fertilizer loss (Liu et al., 2018). After rotary tillage, paddy fields typically require a resting period before rice transplanting or sowing, during which fertilizer dissolves and is subject to volatilization and leaching (Ding et al., 2023), leading to nitrogen losses (Alam et al., 2020).

Side-deep banding fertilizer method is a method that places fertilizer in the deeper soil layers near the crop root zone (Li et al., 2022a; Hou et al., 2023). This method is generally synchronized with rice sowing or transplanting, thereby reducing field operations and labor intensity (Min et al., 2021; Zhong et al., 2021). Research by (Pan et al., 2017) showed that mechanical deep nitrogen fertilizer application, compared with manual surface broadcasting, increased NUE, and grain yield, while also improving economic benefits. Similar benefits were also observed by (Yao et al., 2018). However, side-deep fertilizer application still faces challenges, particularly in paddy fields. One common issue is the susceptibility of side-deep fertilizer machinery to clogging, primarily due to the high soil moisture content in rice fields, which causes fertilizer particles to absorb moisture and agglomerate, leading to blockages in transport pipes or outlets (Tang et al., 2021; Wang et al., 2022). Additionally, soil often adheres to fertilizer machinery components due to the wet or heavy soils, further obstructing fertilizer outlets (Luo et al., 2022). These issues can reduce operational efficiency, potentially damage machinery, and ultimately hinder rice production.

Layered fertilizer method is an approach designed to reduce nutrient losses and optimize fertilizer utilization rate (Liu et al., 2024). The core principle lies in layered fertilization in deep soil layers based on the root distribution and nutrient requirements of crops (He et al., 2024), ensuring effective nutrient absorption throughout different growth stages (Liu et al., 2023a) (Wu et al., 2023). demonstrated that compared to manual surface broadcasting, layered fertilizer application balances nutrient distribution in the root zone, effectively enhances nitrogen use efficiency, and increases crop yields. However, this method demands higher performance from the machinery used. Considering the advantages and disadvantages of the aforementioned fertilizer application methods, a novel nitrogen fertilizer application method, named full root-zone fertilizer application method (RF), has been applied during rice transplanting. This method spreads fertilizer in bands on the soil surface beside the rice seedlings. The fertilizer outlet is suspended in the air and does not touch the paddy field soil. Subsequently, the rear mounted tilling wheels mix the fertilizer with the soil, distributing it in layers within the soil surrounding the root-zone of the seedlings. The machine was developed by the engineering college of South China Agricultural University, named full root-zone fertilizer machine. The RF method achieves the effects of stratified deep fertilizer application while addressing the clogging issues caused by contact between fertilizer and paddy soil. However, there is little research on the actual agronomic effect of this method.

To explore the mechanism of the RF method on rice productivity, this study conducted three repeated experiments in South China (Zhaoqing, Jiangmen) for two years using the developed full root-zone machine. The objectives of this study were to: 1) investigate the impact of the RF method on rice physiological characteristics (root system, tiller number, soil and plant analyzer development (SPAD) values, and antioxidant enzyme activity); 2) explore the impact of the RF method on rice yield, NUE, and economic benefits; 3) compare the RF method with manual broadcasting and deep-side fertilizer application methods to study the effects of different nitrogen fertilizer approaches on rice production. This research aimed to provide effective nitrogen fertilizer application strategies for enhancing rice production.

## 2 Materials and methods

### 2.1 Description of the machine and experimental site

#### 2.1.1 Fertilizer machine

**Figure 1:**
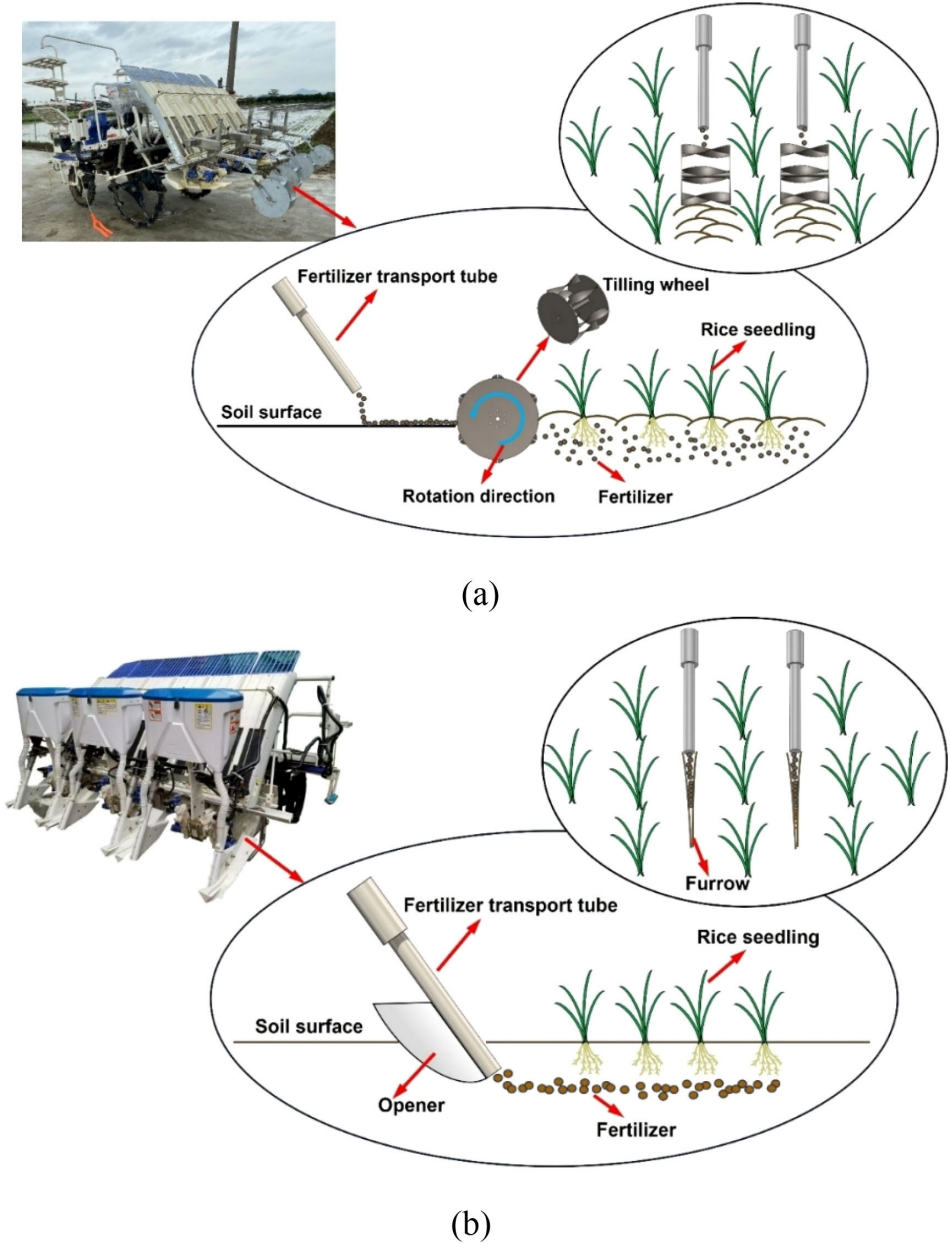
Schematic diagram of the operation of two fertilizer machines, including side section view and top view: **(a)** full root-zone fertilizer machine; **(b)** deep-side banding fertilizer machine.

The full root-zone fertilizer machine and deep-side banding fertilizer machine (*HUNAN LONGZHOU FARM EQUIPMENT HOLDINGS CO., LTD.*) were used in the field experiments. The full root-zone fertilizer machine spreads fertilizer in bands on the soil surface beside the seedlings through the fertilizer transport tube while transplanting. The rear mounted tilling wheels go 5–15 cm deep into the soil layer, and mix the fertilizer and soil, ensuring that the fertilizer is evenly distributed in the soil layers of varying depths near the root systems of the rice seedlings (**Figure 1a**). The fertilizer transport tube and tilling wheels are operated between the seedling rows to avoid damaging the seedlings. The deep-side banding fertilizer machine opens the soil on the side of the seedlings using furrow openers, and deeply places the fertilizer into the furrow for 5-15cm through the fertilizer transport tube while transplanting (**Figure 1b**). The fertilizer transport tube and the furrow openers are operated in the middle of the rice row to avoid damaging the seedlings.

#### 2.1.2 Experimental sites

**Figure 2:**
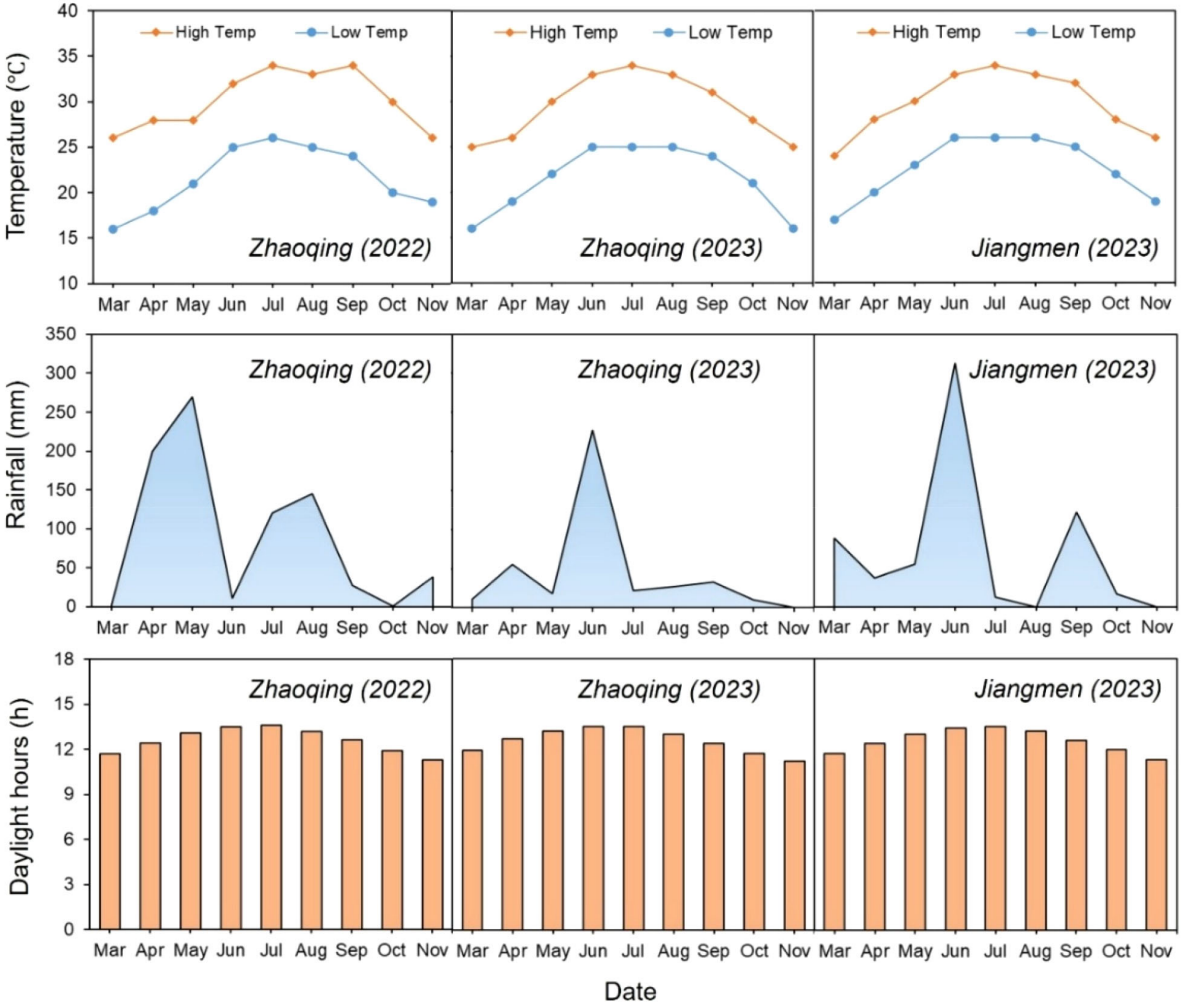
Weather data at the experimental sites.

The experiments for late rice season in 2022 was conducted at Shapu Farm, Dinghu District, Zhaoqing City, Guangdong Province (23°15’N, 112°65’E). The experiment for early rice season in 2023 was carried out at two locations: Shapu Farm, and the Agricultural Science Research Institute in Jiangmen, Guangdong Province (22°77’N, 112°97’E). The soil texture at the Zhaoqing and Jiangmen experimental sites were light loam and light clay, with an organic matter content of 17.43 and 32.78g kg^-1^, total nitrogen content of 0.98 and 1.82 g kg^-1^, alkali-hydrolyzed nitrogen content of 86.49 and 151.97 mg kg^-1^, available phosphorus content of 21.59 and 17.56 mg kg^-1^, available potassium content of 72.94 and 217.97 mg kg^-1^, and PH values of 7.30 and 6.24, respectively. Noteworthy, both the Jiangmen and Zhaoqing experimental sites share a similar subtropical monsoon climate. It is characterized by consistently high annual temperatures, distinct hot summers, mild winters, and relatively stable year-to-year temperature fluctuations. Rainfall distribution is highly seasonal, with the majority concentrated in the April-September period, while sunshine hours correspondingly vary with the precipitation patterns. **Figure 2** shows the detailed weather data of two sites during rice growth period in 2022 and 2023.

### 2.2 Experimental design

Three rice types: Huahang 72, Huahang 57, and Lixiangzhan were used in late rice season experiment in 2022 and early rice season experiments in 2023, respectively. All three rice types are the conventional high-quality indica rice varieties widely used in South China. In all experiments, rice seedlings were mechanically transplanted using the equipment specified in Section 2.1.1. The configuration was set to a row spacing of 30 cm, a plant spacing of 18 cm, and a transplanting depth of 2 cm, corresponding to a planting density of 1.852×10^5^ holes ha^−1^.

Nitrogen fertilizer is the main factor in increasing rice yield. Nitrogen was supplied through a combination of Rainbow compound fertilizer (N: P: K = 24: 7: 19) and urea (46% nitrogen content), a strategy tailored to the soil nutrient characteristics and local agronomic guidelines. This approach ensured the supplementation of phosphorus and potassium. Four fertilizer application treatments were implemented for each experiment: blank control, no nitrogen fertilizer applied throughout the growth cycle (C); full root-zone fertilizer application (RF); deep-side fertilizer application (DF); manually broadcasting fertilizer application (BF). Based on the agronomic fertilizer application requirements for the South China region, a total nitrogen rate of 180 kg ha^−1^ was applied throughout the rice growth cycle. In treatments RF, DF, and BF, the basal nitrogen fertilizer of 100 kg ha^−1^ was applied while transplanting, followed by the topdressing of 80 kg ha^−1^ nitrogen applied manually within 15–20 days after transplanting. **Table 1** shows the summary of the nitrogen fertilizer application treatments in experiments. Phosphate and potassium fertilizers were provided by Rainbow compound fertilizer, P_2_O_5_ and KCl, ensuring same application of phosphate and potassium fertilizers at different rice growth stage for all treatment. 90 kg ha^-1^ of P and 150 kg ha^-1^ of K were applied throughout the whole rice growth period. For base fertilizer, RF, DF and BF treatments applied 30 kg ha^-1^ of P and 80 kg ha^-1^ of K using compound fertilizers, and C treatment applied P_2_O_5_ and KCl to ensure consistent P and K rates. For late top dressing, all treatments were treated with P_2_O_5_ and KCl with 60 kg ha^-1^ of P and 70 kg ha^-1^ of K.

**Table 1:** Summary of the nitrogen fertilizer application treatments in experiments.

Treatment	Total N rate (kg ha^-1^)	Basal nitrogen fertilizer	Top dressing nitrogen fertilizer	
		N rate (kg ha^-1^)	N rate (kg ha^-1^)	Application method
C	–	–	–	–
RF	180	100	80	Broadcasting
DF	180	100	80	Broadcasting
BF	180	100	80	Broadcasting

C, no nitrogen fertilizer applied throughout the growth cycle; RF, full root-zone fertilizer application; DF, deep-side fertilizer application; BF, manually broadcasting fertilizer application.

In each experiment, each treatment was repeated in three plots, with a total of 12 plots. Each plot measured 10 m × 20 m. Plots were randomly distributed in the field. Ridges were constructed between the plots and covered with plastic film to prevent runoff. The seeding, transplanting, and harvesting dates for the experiments are summarized in **Table 2**.

**Table 2:** Seeding, transplanting, and harvesting times of 2022 early rice season and 2023 late rice season.

Year	Location, season	Seeding time	Transplantation time	Harvest time
2022	Zhaoqing, late season	Jul. 16	Aug. 5	Nov. 12
2023	Zhaoqing, early season	Mar. 3	Mar. 28	Jul. 14
2023	Jiangmen, early season	Mar. 5	Mar. 29	Jul. 16

All plots were managed uniformly according to local agronomic practices. Water management employed intermittent irrigation to maintain alternating wet and dry conditions. Weed control integrated mechanical methods with selective herbicides, while pests and diseases were managed via precise pesticide applications guided by field monitoring.

### 2.3 Measurements

Some data of rice plants under different treatments were measured at four rice growth stages: late tillering stage (TS), booting stage (BS), heading stage (HS), and maturity stage (MS). Additionally, theoretical rice yields were measured for all treatment plots, and nitrogen absorption was assessed to calculate NUE at MS stage.

#### 2.3.1 Physiological characteristics of rice plants and root

At four growth stages (TS, BS, HS, MS), ten fixed data collection points were randomly selected and marked at first measuring time for each plot. The tiller number was manually recorded (number of effective tillers collected at maturity stage). The SPAD values were calculated by measuring chlorophyll content of uppermost plant leaves using a portable chlorophyll testing instrument (*SPAD-502 Plus, Konica Minolta*), repeating the test three times for this value and taking the average. Root characteristics were measured at the TS stage. Ten representative plant samples were randomly collected in each treatment plot. To ensure the complete preservation of rice roots, the plant sample was taken as the center during excavation, the distance around was 15cm, and the excavation depth was 35cm. The roots were carefully washed to ensure their integrity and separated. The root scanning instrument (*ScanMaker-i800 Plus, MICROTEK*) were used for measuring the root characteristics data including average root length (RL), average diameter (RD), and total root surface area (RA). The root dry matter weight (RW) was recorded by drying at 80°C for 48 h using a dryer.

#### 2.3.2 Antioxidant enzyme activity

At the aforementioned growth stages, ten uppermost leaf samples of plant were collected from each plot and stored in low temperature incubator to measure the antioxidant enzyme activity of plants. Superoxide dismutase (SOD), and peroxidase (POD) of plants under different fertilizer application treatments were measured in accordance with the approaches (Pan et al., 2013) and (Pan et al., 2017) used.

#### 2.3.3 Aboveground dry matter weight, rice yield and its composition

At each of the growth stages, ten sample plants were randomly selected from each plot. The plants were washed and their roots were removed. The plants were dried at 80°C for 48 hours to estimate aboveground dry matter weight. At the MS stage, plant samples were collected from three randomly selected areas of 1 m2 per plot to determine the theoretical rice yield. The rice yield components, including the effective panicle number (EP), grains per panicle (GP), seed setting rate (SR), and 1000-grain weight (GW), were measured manually and using an intelligent seed testing machine (Guangdong Jiuzhou Zhinong Technology Co., Ltd) (Liu et al., 2023b). The theoretical rice yield (RY) was calculated by Equation 1 below:

(1) $$\begin{array}{c} R_{Y}=E_{P}*G_{P}*S_{R}*G_{W}*10^{-6} \end{array}$$

#### 2.3.4 Nitrogen use efficiency

Ten holes plant samples were selected to separate into three parts: stems, leaves, and grains from each plot at MS stage. All parts were dried at 105°C for 1h using a dryer for enzyme activity inactivation treatment, followed by drying at 80°C for 48 h. Then, the dried samples were sieved into fine powder. Refer to the Kjeldahl method used by (Pan et al., 2017), the plant nitrogen content (NC) was measured. Nitrogen absorption (NA) and NUE were then calculated. NUE included nitrogen physiological efficiency (NPE), nitrogen agronomic efficiency (NAE), and nitrogen recovery efficiency (NRE). The (Equations 2–5) for all calculations are shown below:

(2) $$\begin{array}{c} N_{A}=A_{W}*N_{C} \end{array}$$

(3) $$\begin{array}{c} N_{AE}=\frac{\left(R_{Y}-R_{Y0}\right)}{\left(N_{A}-N_{A0}\right)} \end{array}$$

(4) $$\begin{array}{c} N_{PE}=\frac{R_{Y}-R_{Y0}}{F_{N}} \end{array}$$

(5) $$\begin{array}{c} N_{RE}=\frac{\left(N_{A}-N_{A0}\right)}{F_{N}} \end{array}$$

where *A_W_* represents the aboveground dry matter weight; *N_C_* represents the plant nitrogen content; *R_Y_* and *R_Y0_* represent the rice yield in the nitrogen-fertilized plot and non-fertilized plot, respectively; *N_A_* and *N_A0_* represent the nitrogen absorption in the nitrogen-fertilized plot and non-fertilized plot, respectively. *F_N_* represents the amount of nitrogen fertilizer rate applied.

#### 2.3.5 Economic benefits

To investigate the impact of fertilization methods on economic benefits, this study recorded and collected the total production cost, total sales value, and net income of rice under different fertilization treatments. These data determine whether the fertilization method can be widely accepted and promoted by farmers (Pan et al., 2017). The total production cost of rice includes the variable cost (rice seeds, fertilizers, pesticides, machinery usage, and labor) and the fixed cost (agricultural insurance). The total rice value refers to the product of rice yield and unit price. Net income refers to the difference between the total sales value of rice and the total production cost.

### 2.4 Data analysis

*IBM SPSS Statistics 27* and *Excel 2019* were used for data analysis and chart drawing. To control the impact of climate and space environment, the experimental years and field groups were included in the statistical model as random effects. Multi-way analysis of variance (ANOVE) was used to evaluate the main effects of rice type, treatment and their interaction on rice measurement indexes parameters, so as to clearly consider the differences of rice types and the specificity of rice types’ response to treatment. The least significant difference (LSD) test was used to determine the differences between the means under different treatments at the 0.05 probability level. *RStudio 4.1.1* was used to process the pearson correlation analysis between rice production indicators at the 0.01 and 0.05 probability levels.

## 3 Results

### 3.1 Physiological characteristics

#### 3.1.1 Tiller number

**Figure 3:**
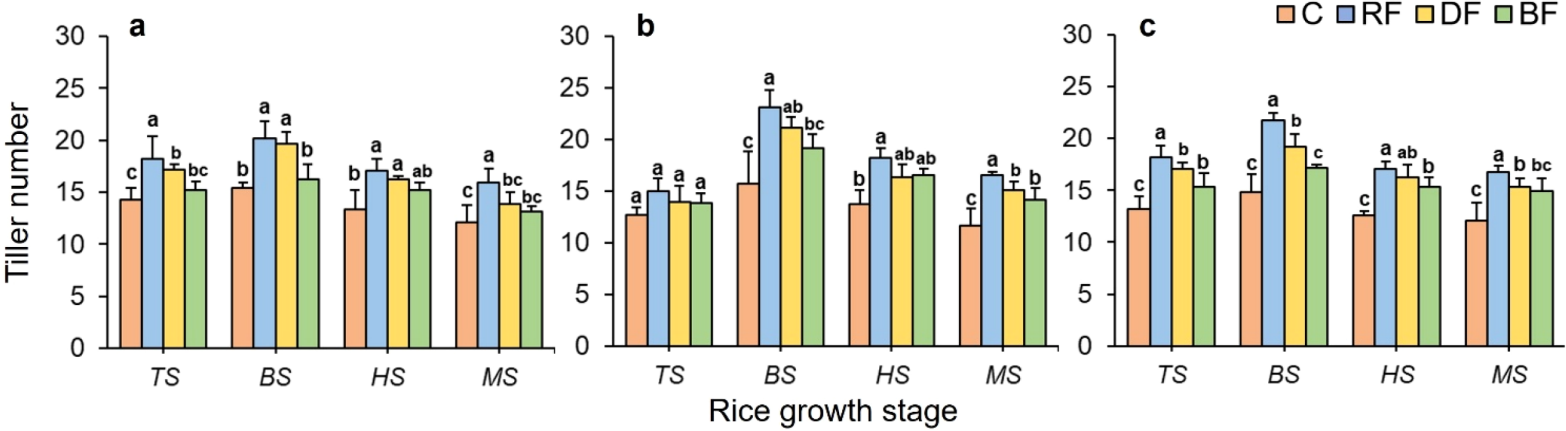
Effects of different treatments on tiller number for three rice types: **(a)** Huahang 72, **(b)** Huahang 57, **(c)** Li Xiangzhan at four rice growth stages: tillering stage (TS), booting stage (BS), heading stage (HS), and maturity stage (MS); C, no nitrogen fertilizer applied throughout the growth cycle; RF, full root-zone fertilizer application; DF, deep-side fertilizer application; BF, manually broadcasting fertilizer application. Means marked with different letters show significant differences (P < 0.05).

**Figure 3** shows the effects of different treatments on the tiller number of rice plant at various growth stages. For all rice types, the tiller number exhibited a trend of increasing from the TS stage to BS stage and then decreasing from the BS stage to the MS stage under all fertilizer application treatments. Peak values always appeared at the BS stage, while the lowest values occurred at the MS stage. Significant differences were shown in the tiller number among different treatments at various growth stages. The mean tiller number under the RF treatment was the highest for the rice types of Huahang72 and Li Xiangzhan at the TS stage, compared with the BF treatment, increasing by 12.08% to 21.37%. At the middle and later rice growth stage, the tiller number under the RF treatment was significantly higher than other treatments across all rice types, and maintain the same trend as the early stage. There was no significant difference in tiller number between RF treatment and DF treatment at all growth stages of different rice types, but the mean performance was higher under RF treatment. These findings indicate that the RF method significantly promotes tillering ability compared to traditional broadcasting method, and has similar effects with DF method.

#### 3.1.2 SPAD

**Figure 4:**
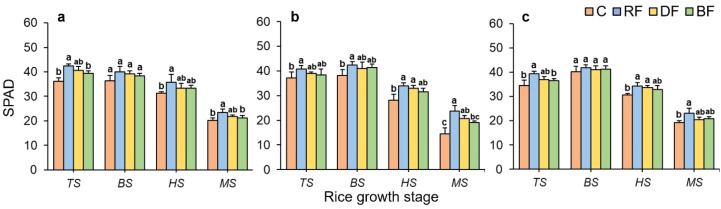
Effects of different treatments on SPAD values for three rice types: **(a)** Huahang 72, **(b)** Huahang 57, **(c)** Li Xiangzhan at four rice growth stages. Means marked with different letters show significant differences (P < 0.05).

For Huahang57 and Li Xiangzhan, the SPAD values under different treatments both showed highest values at the BS stage. During the later stages, SPAD values gradually decreased, and at the MS stage, it is significantly lower than other stages. For the rice type of Huahang72, the SPAD values under RF treatment were significantly higher than BF and C. (**Figure 4a**). For the rice types of Huahang57 and Li Xiangzhan, significant differences in SPAD values were observed at the early and later stages under different treatments (**Figures 4b, c**). Notably, the mean SPAD values under RF treatment resulted in relatively higher values for all rice types at the MS stage. This suggests that rice plants under the RF treatment had higher growth vigor compared to other fertilizer application treatments during the same period and exhibited a longer growth cycle.

### 3.2 Rice root characteristics

**Figure 5:**
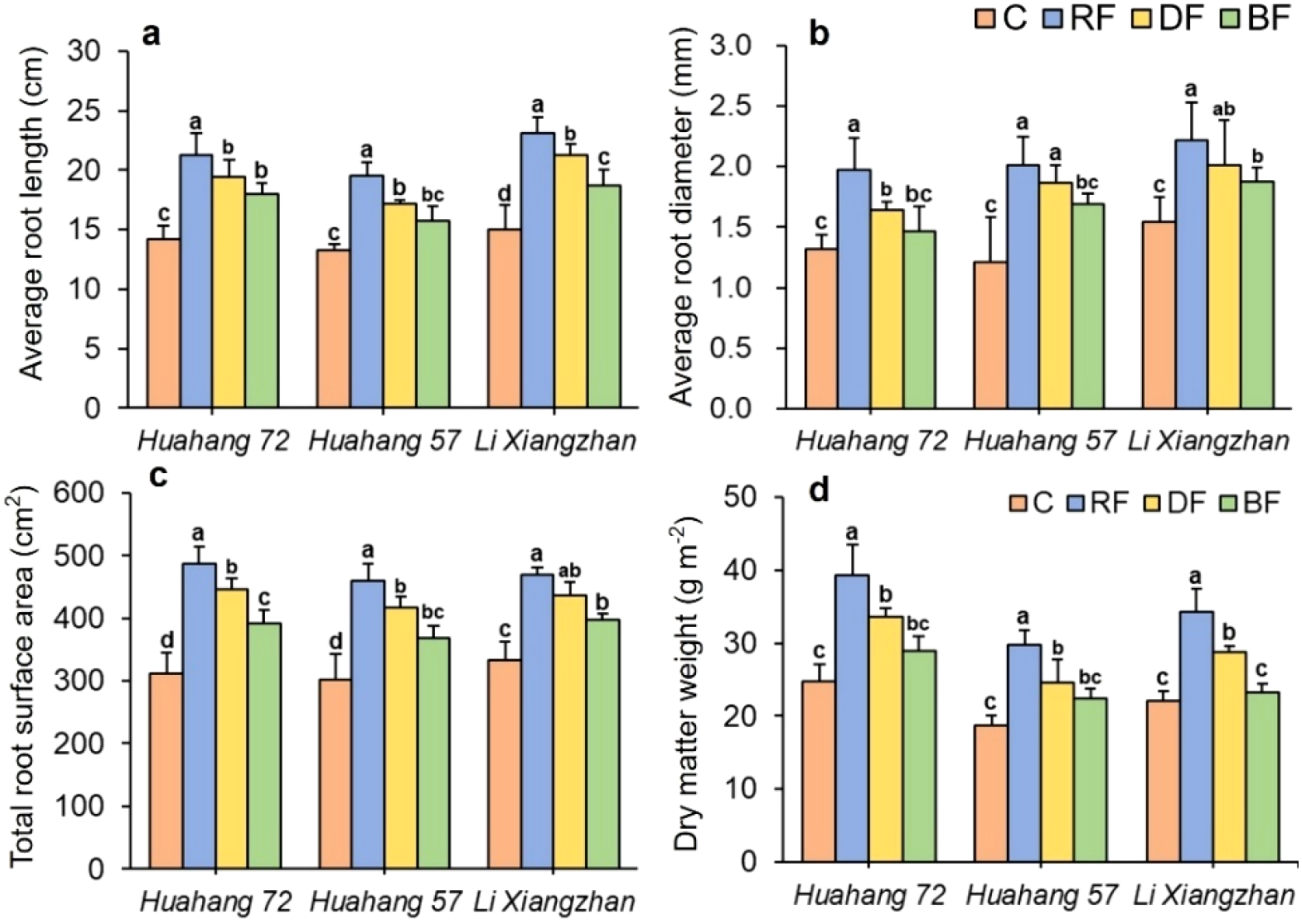
Effects of different treatments on rice root characteristics: **(a)** average root length, **(b)** average root diameter, **(c)** total root surface area, and **(d)** root dry matter weight for three rice types at TS stage. Means marked with different letters show significant differences (P < 0.05).

Fertilizer application treatments had significant effects on the rice root characteristics at the TS stage, including the average root length (RL), average root diameter (RD), total root surface area (RA), and root dry matter weight (RW) (**Figure 5**). The rice type of Li Xiangzhan exhibited the longest RL compared to other types. For all rice types, the RF treatment had the best rice root characteristics, resulting in an increasing 24.41% of RL, 34.93% of RD, and 25.22% of RA, respectively, compared to BF treatment. There was no significant difference in RA, RD, and RA under RF treatment compared to DF treatment. But overall, the mean values were higher under RF processing. Additionally, the RW was the highest under the RF treatment of rice types of Huahang72 and Li Xiangzhan, with 17.03% to 20.81% and 32.81% to 47.46% increases compared to the DF and BF treatments respectively. The rice type of Huahang72 exhibited the highest RL. These findings indicate that the RF method effectively promotes early root growth compared with traditional broadcasting method, and has similar effects and even shows better root system performance compared with the DF method.

### 3.3 Antioxidant enzyme activity in rice plants

**Figure 6:**
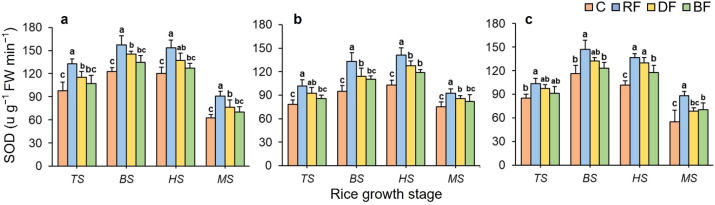
Effects of different treatments on SOD activity for three rice types: **(a)** Huahang 72, **(b)** Huahang 57, **(c)** Li Xiangzhan at four rice growth stages. Means marked with different letters show significant differences (P < 0.05).

**Figure 6** illustrates the impacts of different treatments on SOD activity at various growth stages of rice plant. For all rice types, SOD activity both had a trend of first increasing and then decreasing during the rice growth stages, peaking at the middle rice growth stage, and reaching the lowest values at the MS stage. The SOD activity under different treatments showed the significant differences, with the overall trend of mean values being RF > DF > BF > C. For all rice types, at all rice growth stages, the SOD activity under the RF treatment both showed the highest value, compared to the DF and BF treatments. Especially, the RF treatment maintained the highest SOD activity of Huahang 72 rice type, with improvements of 20.47% and 29.59% over the DF and BF treatments, respectively at the MS stage.

**Figure 7:**
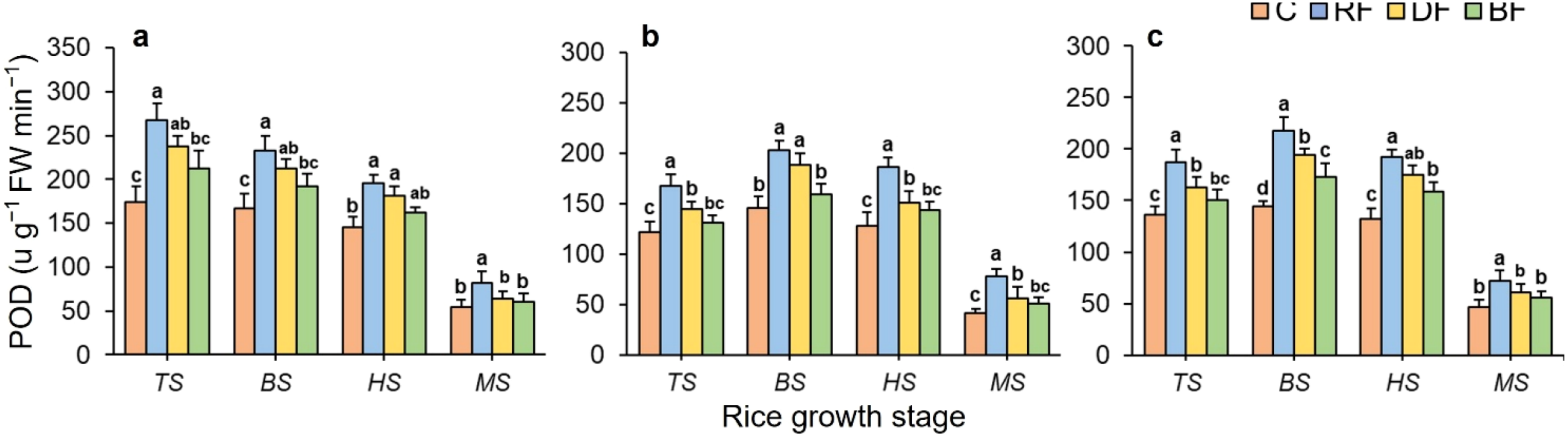
Effects of fertilizer application treatments on POD activity for three rice types: **(a)** Huahang 72, **(b)** Huahang 57, **(c)** Li Xiangzhan at four rice growth stages. Means marked with different letters show significant differences (P <0.05).

For the rice type of Huahang72, the POD activity showed the peak value at the TS stage and gradually declined during later stages (**Figure 7a**). In both early rice seasons of 2023, the POD activity followed a change of first increasing and then decreasing throughout the rice growth stage, with a peak observed at the BS stage (**Figures 7b, c**). The POD activity under different treatments both decreased to their lowest levels at the MS stage for all rice types. The POD activity under different treatments occurred significant differences, with the overall trend being RF > DF > BF > C. At the TS stage, the POD activity of rice plants under the RF treatment of Huahang 57 and Li Xiangzhan rice types were highest, increasing by 12.74% to 15.47% and 24.32% to 27.25% compared to the DF and BF treatments, respectively. Similarly, at the middle rice growth stage, RF treatment resulted in the highest POD activity. Even at the MS stage, the POD activity under RF treatment maintained the highest, showing increases of up to 52.56% compared to the DF and BF treatments. These results demonstrate that the RF method significantly enhances the antioxidant enzyme activity in rice plants, effectively prolonging the growth cycle.

### 3.4 Aboveground dry matter weight, rice yield and its components

**Figure 8:**
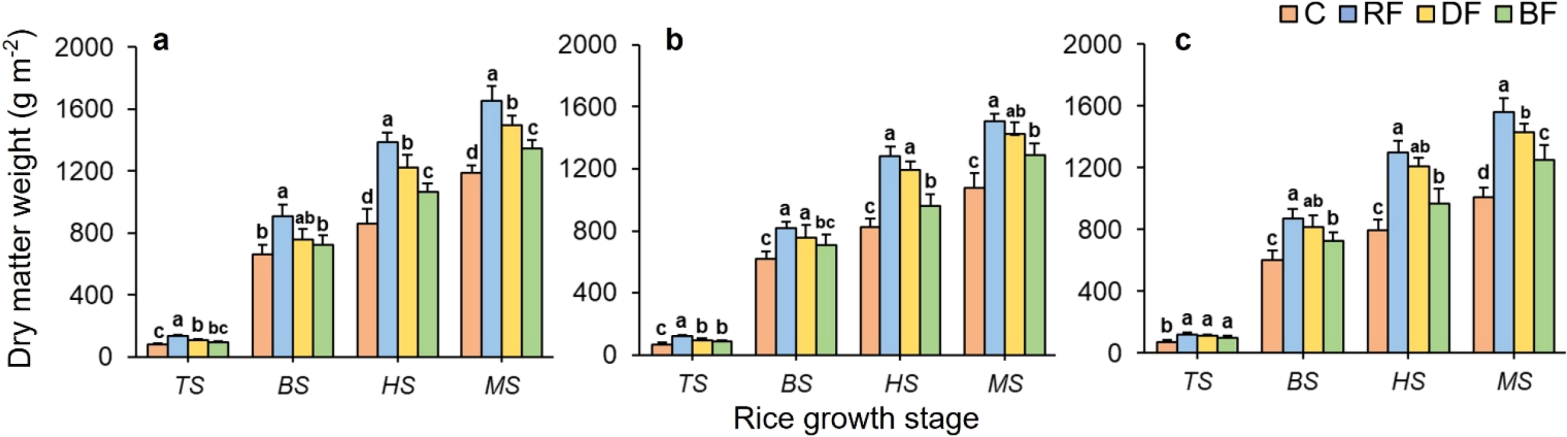
Effects of different treatments on aboveground dry matter weight for three rice types: **(a)** Huahang 72, **(b)** Huahang 57, **(c)** Li Xiangzhan at four rice growth stages. Means marked with different letters show significant differences (P < 0.05).

Significant differences in aboveground dry matter content were observed under different treatments (**Figure 8**). The aboveground dry matter weight of Huahang72 and Huahang57 was highest under the RF treatment at the TS stage, showing increases of 22.02% to 28.56% and 35.63% to 46.42% compared to the DF and BF treatments, respectively. No significant difference under different treatments for rice type of Li xiangzhan. At the later rice growth stages, the aboveground dry matter weight had an overall trend being RF> DF > BF > C. At the MS stage, the aboveground dry matter weight under the RF treatment was higher than others, with increases from 6.12% to 25.22% compared to the DF and BF treatments, respectively. Notably, there is no significant difference between RF and DF treatments, but the mean performance is higher under RF treatment.

Analysis of variance revealed that rice types had a significant effect on R_Y_ (P< 0.05) and highly significant effects on the G_P_, S_R_, and G_W_ (P< 0.01), no significant effect on the E_P_ (P > 0.05). Treatment had highly significant effects on E_P_, G_P_, S_R_, and R_Y_ (P< 0.01), no significant effect on G_W_ (P > 0.05). Also, no significant effect on above parameters was shown in the interaction between rice type and treatment (**Table 3**). For some rice types, significant differences were observed in rice yield and its components (except G_W_) under different fertilizer application treatments. The RF treatment resulted in the highest mean values of yield components. The E_P_ under RF treatment of Huahang72 and Huahang57 rice types were increased compared to BF treatment, with 17.02% and 21.37%, respectively. Also, up to 7.08% increase in the G_P_ and up to 4.22% increase in S_R_ were observed under the RF treatment compared to other two treatments. More importantly, the R_Y_ was maximized under RF treatment, with increases of 13.87% to 21.32% and 17.94% to 33.41% compared to DF and BF.

**Table 3:** Effect of different treatments on the effective panicle number (*E_P_*), grains per panicle (*G_P_*), seed setting rate (*S_R_*), and 1000-grain weight (*G_W_*), and rice yield (*R_Y_*) for three rice types in experiments.

Year	Rice type	Treatment	*E_P_* (10^4^ ha^-1^)	*G_P_*	*S_R_* (%)	*G_W_* (g)	*R_Y_* (t ha^-1^)
2022	Huahang72	C	261.15c	118.12c	86.17b	21.04a	5.58d
		RF	341.85a	130.06a	89.47a	21.51a	8.54a
		DF	298.85b	127.20ab	87.17ab	21.24a	7.04b
		BF	281.65b	122.67b	86.87b	20.95a	6.40c
2023	Huahang57	C	251.55c	138.22c	83.27c	17.59a	5.09d
		RF	354.75a	151.33a	87.20a	18.01a	8.29a
		DF	324.65ab	145.14ab	85.23b	17.69a	7.10b
		BF	303.15b	141.33bc	83.67c	17.51a	6.27c
2023	Lixiangzhang	C	254.12c	117.67b	82.83a	19.21a	4.77c
		RF	350.74a	125.67a	83.47a	19.08a	7.02a
		DF	321.32ab	121.60ab	82.91a	19.23a	6.16b
		BF	312.91b	120.33b	82.67a	19.11a	5.94b
ANOVA	Rice type		ns	**	**	**	*
	Treatment		**	**	**	ns	**
	Rice type * Treatment		ns	ns	ns	ns	ns

C, no nitrogen fertilizer applied throughout the growth cycle; RF, the full root-zone fertilizer application; DF, the deep-side fertilizer application; BF, the manually broadcasting fertilizer application. ANOVA represents the analysis of variance in rice variety, treatment, and their interaction. ns represents a non-significant difference. * and ** represent significant differences at 0.05 and 0.01 probability levels, respectively. Means marked with different letters show significant differences (P < 0.05).

### 3.5 Nitrogen use efficiency

Nitrogen use efficiency plays a key role in rice production (Zhang et al., 2022). **Table 4** displayed the effects of different treatments on nitrogen absorption (N_A_) and NUE. Analysis of variance indicated that rice types had a highly significant impact on nitrogen absorption (P< 0.01), and no significant impact on nitrogen use efficiency (P > 0.05). Treatments had highly significant impacts on both nitrogen absorption and NUE (P< 0.01). No significant effect on nitrogen absorption and NUE was shown in the interaction between rice type and treatment (P > 0.05). For all rice types, significant differences in nitrogen absorption were observed under different fertilizer application treatments, with RF showing the highest mean values, increasing by 11.31% to 15.63% and 31.44% to 41.37% compared to DF and BF, respectively. The mean values of nitrogen agronomic use efficiency (N_AE_) were also the highest under RF. No significant differences were observed in nitrogen physiological use efficiency (N_PE_) among the treatments. The mean values of nitrogen recovery use efficiency (N_RE_) were highest under the RF treatment, increasing by 28.88% to 52.77% and 149.97% to172.56% compared to DF and BF.

**Table 4:** Effect of different treatments on nitrogen absorption (*N_A_*), nitrogen agronomic efficiency (*N_AE_*), nitrogen physiological efficiency (*N_PE_*), and nitrogen recovery efficiency (*N_RE_*) for three rice types.

Year	Rice type	Treatment	*N_A_* (kg ha^-1^)	*N_AE_* (kg kg^-1^)	*N_PE_* (kg kg^-1^)	*N_RE_* (%)
2022	Huahang72	C	129.72d	–	–	–
		RF	218.62a	16.41a	36.34a	45.15a
		DF	189.08b	8.05b	27.32a	29.55ab
		BF	163.05c	4.53b	27.04a	16.75b
2023	Huahang57	C	98.13d	–	–	–
		RF	172.11a	17.80a	43.75a	40.67a
		DF	154.62b	11.17ab	35.39a	31.56ab
		BF	130.94c	6.53b	43.74a	14.92b
2023	Li Xiangzhang	C	109.59d	–	–	–
		RF	207.72a	12.47a	24.10a	51.75a
		DF	179.95b	7.72ab	20.99a	36.78b
		BF	146.94c	6.53b	31.58a	20.70c
ANOVA	Rice type		**	ns	ns	ns
	Treatment		**	**	ns	**
	Rice type * Treatment		ns	ns	ns	ns

C, no nitrogen fertilizer applied throughout the growth cycle; RF, the full root-zone fertilizer application; DF, the deep-side fertilizer application; BF, the manually broadcasting fertilizer application. ANOVA represents the analysis of variance in rice variety, treatment, and their interaction; ns represents a non-significant difference. * and ** represent significant differences at 0.05 and 0.01 probability levels, respectively. Means marked with different letters show significant differences (P < 0.05).

### 3.6 Economic benefits

**Table 5** shows that the total production costs under BF treatment is the highest, increasing by 1.8% compared to RF and DF treatments. This is mainly due to the fact that the fertilization method requires more labor costs. The total rice value under RF treatment was the highest, increasing by 17.43% and 22.73% compared to DF and BF treatments. The net income also showed the highest performance under RF processing, with an increase of 31.77% and 45.33% compared to DF and BF processing. These findings indicate that RF and DF treatments reduce labor costs compared to BF treatment, while also increasing net income by improving rice yields, with RF treatment showing the highest level of net income.

**Table 5:** The total production cost, total value, and net income of rice under different treatments.

Treatment	Total production costs (CNY ha^-1^)						Total rice value (CNY ha^-1^)			Net income (CNY ha^-1^)
	Seed	Fertilizer	pesticide	Fertilizer application	Other expenses	Total cost	R_Y_ (t ha^-1^)	Price	Total value	
RF	1312.5	2937.5	1725	195	44832	11002	7.95	3.6	28620	17618
DF	1312.5	2937.5	1725	195	4832	11002	6.77	3.6	24372	13370
BF	1312.5	2937.5	1725	390	4832	11197	6.20	3.6	23320	12123

Other expenses represent the cost of labor and machines in rotary tillage, management, harvest, and the agricultural insurance. R_Y_ represents the rice yields, which were the average of four rice types in all rice seasons. Price represents the average price of three types of indica rice (CNY kg^-1^), and the data comes from official data from the Guangdong Provincial Agriculture Bureau.

### 3.7 Correlation analysis

**Figure 9:**
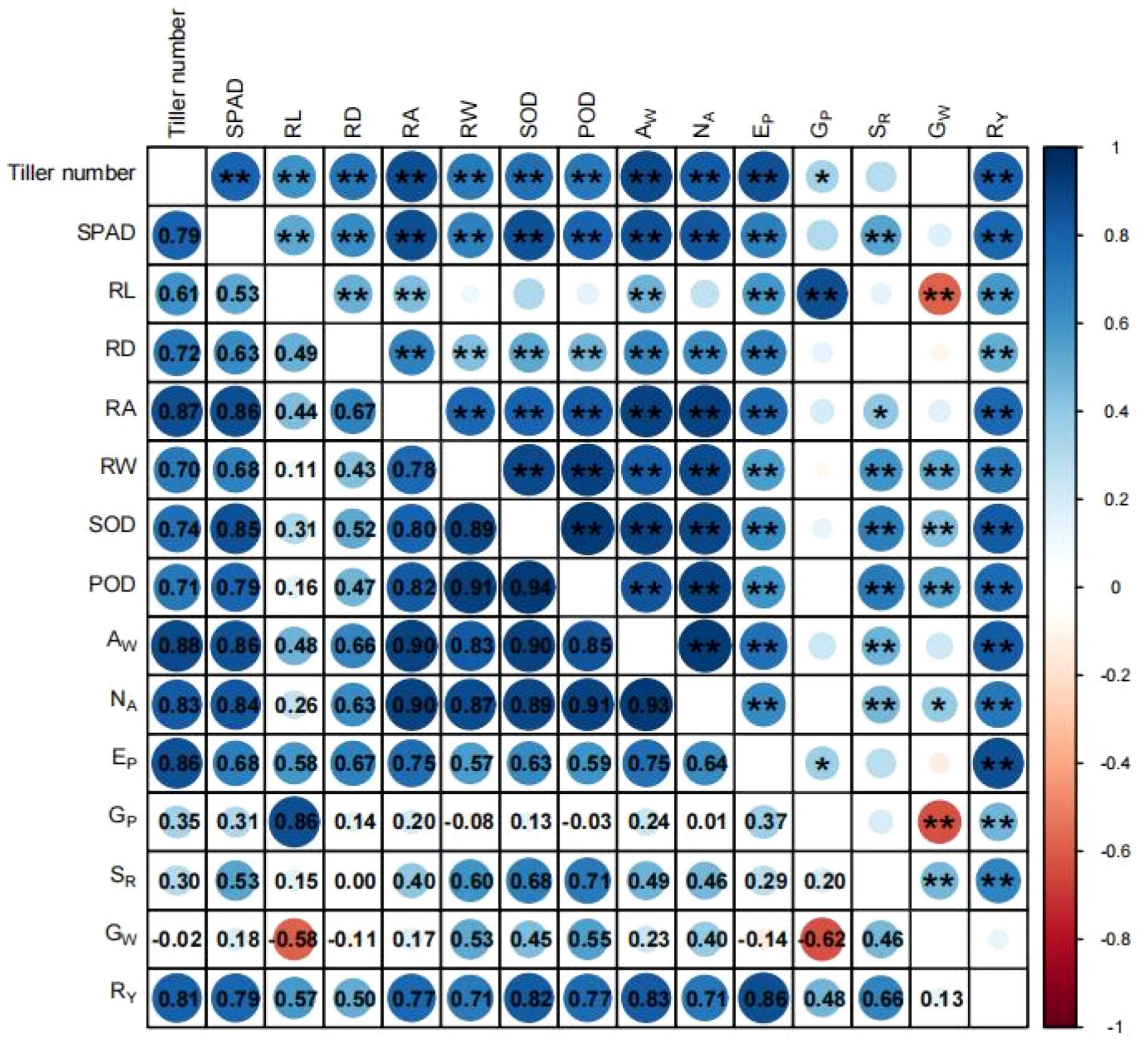
Correlation analysis between rice indicators in 2022 and 2023. Blue represents the positive correlation, and red represents the negative correlation. The numbers in each cell represent the correlation coefficient, and the depth of color represents the strength of correlation. * and ** represent significant correlations at 0.05 and 0.01 probability levels, respectively. RL, average root length; RD, average root diameter; RA, total root surface area; RW, root dry matter weight; A_W_, aboveground dry matter weight of rice plants; N_A_, nitrogen absorption; E_P_, effective panicle number; G_P_, grain number per panicle; S_R_, seed-setting rate; G_W_, 1000-grain weight; R_Y_, rice yield.

Pearson correlation analysis between rice indicators under different treatments in two years are shown in **Figure 9**. Highly significant positive correlations were shown between Rice yield (R_Y_) and nitrogen absorption (N_A_), effective panicle number (E_P_), seed setting rate (S_R_), grain number per panicle (G_P_), antioxidant enzyme activity, and root characteristics (P< 0.01). Both SOD and POD activities were significantly positively correlated with E_P_ S_R_, tiller number, SPAD, average root diameter (RD), total root surface area (RA), and root dry matter weight (RW) (P< 0.05). Average root length (RL) had significant positive correlations with E_P_, G_P_, tiller number, SPA, and aboveground dry matter weight (A_W_) (P< 0.05), but showed a highly significant negative correlation with 1000-grain weight (G_W_) (P< 0.01). RD was significantly positively correlated with N_A_, E_P_, tiller number, SPAD, and A_W_ (P< 0.01). RA showed significant positive correlations with N_A_, E_P_, tiller number, SPAD, and A_W_ (P< 0.01), and had a significant positive correlation with S_R_ (P< 0.05). RW showed highly significant positive correlations with N_A_, E_P_, S_R_, G_W_, tiller number, SPAD, and A_W_ (P< 0.01).

## 4 Discussion

### 4.1 Effects of different fertilizer application methods on physiological characteristics and root characteristics of plants

Fertilizer application methods significantly impact rice growth (Zhuang et al., 2022). The efficient fertilizer application method can effectively improve the physiological characteristics of rice plants (Sui et al., 2013). The machine performs subsoiling and overturning of the soil near the root zone during fertilizer application in the full root-zone fertilizer application method. Subsoiling increases soil porosity (He et al., 2019), effectively improving soil looseness and aeration (Ning et al., 2022). This provides more space for root growth (Hamilton et al., 2019), promoting deeper root development, and increases the contact area between crop roots and soil, thereby enhances the absorption capacity of roots for nutrients (Cai et al., 2014; Lynch et al., 2022). In this study, the full root-zone fertilizer application method exhibited superior rice root characteristics, including average root length (RL), average root diameter (RD), total root surface area (RA), and root dry matter weight (RW) compared to surface broadcasting. It also has similar effects and even shows better root system performance compared with deep-side fertilizer application method. Both methods have made changes to the soil structure during operation. Rice tiller formation requires a significant amount of nutrients and water (Dou et al., 2016). Robust root systems can absorb more nutrients, activate the division and differentiation of tiller buds, and promote the production of tillering (Fageria, 2007).

Simultaneously, strong roots provide sustained nutritional support for rice tillering, helping to maintain tillering stability (Kameoka et al., 2016). Consequently, compared to surface broadcasting fertilizer application, the full root-zone fertilizer application method achieved the higher tillers of rice plants at the tillering stage and maintained a similar trend in the later growth stages. Although there was no significant difference in tiller number under side deep and full root-zone fertilizer application method, the mean of the latter was higher. This performance is related to the changes in soil looseness and root development status caused by the two methods. This finding is consistent with the research of (Xu et al., 2022), who found that soil subsoiling contributes to an increase in crop tiller numbers. The SPAD value reflects the chlorophyll content of rice leaves (Saberioon et al., 2014), and chlorophyll is closely associated with nitrogen content (Houles et al., 2007). Therefore, the SPAD value is an important indicator for evaluating the nitrogen content status of plant (Yuan et al., 2016). In this study, rice root characteristics were significantly positively correlated with nitrogen absorption (**Figure 9**). Compared to surface broadcasting and deep-side fertilizer application, the full root-zone fertilizer application method significantly increased nitrogen absorption by 11.31% to 41.37% (**Table 4**).

### 4.2 Effects of different fertilizer application methods on antioxidant enzyme activity of rice plant

During the metabolism of rice growth, reactive oxygen species (ROS) are generated (Pandhair and Sekhon, 2006). Under drought, high temperatures, low temperatures, hypoxia, and salt stress conditions, ROS in plants will accumulate excessively (Zhang et al., 2020), leading to protein oxidation, disruption of gene expression and cell division, and damage to organelles (Garcia-Caparros et al., 2021), thereby impairing plant growth. Antioxidant enzymes is a central key in removing ROS in plants (Kapoor et al., 2019). Through oxidative stress signaling pathways, plants activate transcription factors such as APS/ERF, WRKY, and NAC to regulate the expression of antioxidant enzyme-related genes (Gulzar et al., 2021). Antioxidant enzymes, such as SOD, POD, and catalase (CAT) collaboratively eliminate ROS, ensuring the normal growth of rice plants (Sasi et al., 2021). Research by (Balliu et al., 2021) found that well-aerated soil environments can alleviate oxidative stress in roots, as improved soil structure promotes the development and growth of plant root systems in deep soil layers (Lynch et al., 2022). Deeper root development allows roots to absorb more water and nutrients, effectively mitigating oxidative stress and providing positive feedback for antioxidant enzyme activity (Ahanger et al., 2017). In this study, RD, RA, and RW both had significantly positive correlations with SOD and POD activities, respectively. The antioxidant enzyme activities under the full root-zone fertilizer application method were significantly higher than the broadcasting fertilizer application method, and there was no significant difference compared to side deep fertilizer application method, but the mean was higher. Moreover, high antioxidant enzyme activity can avoid oxidative damage to tiller buds, ensuring their normal division and development (Dwivedi et al., 2019), and providing sufficient nutrient support for tillering growth (Li et al., 2020). Obviously, the trend of tiller number changes under different methods is consistent with the activity of antioxidant enzymes. According to the research of (Ru et al., 2023), during the grain-filling stage, root functionality and antioxidant enzyme activity collectively enhance nitrogen transport and photosynthetic efficiency. In this study, compared to other fertilizer application methods, the mean SPAD value under the full root-zone fertilizer application method was highest at the late stage. Additionally, the seed setting rate under this method was significantly higher than under other fertilizer application methods, attributed to the high antioxidant enzyme activity that mitigated oxidative damage during grain filling (Wang et al., 2014).

### 4.3 Effects of fertilizer application methods on rice yield and economic benefits

The method of nitrogen fertilizer application has a significant impact on rice yield (Zhou et al., 2021). The rice yield (R_Y_) under full root-zone fertilizer application method had significantly increases, up to 33.41%, compared to broadcasting fertilizer application method. Notably, E_P_, G_P_, G_W_, and S_R_ are the primary factors directly influencing the R_Y_ (Yang et al., 2021). Compared with the broadcasting fertilizer application method, the full root-zone fertilizer application method resulted in the higher E_P_, G_P_, and S_R_. This can be explained as follows: The full root-zone fertilizer application method effectively improved soil structure, improving soil oxygen availability, and enhancing deeper root system development (Jin et al., 2017). Meanwhile, Deeper root development enhancing root nitrogen absorption capacity (Lynch et al., 2022). This contributed to improved tillering at the early stages and facilitated spikelet differentiation at middle stages (Cai et al., 2014), thereby increasing the grain number per panicle. Moreover, tiller number directly impacts effective panicle number (Pasuquin et al., 2008). Thus, enhanced tiller number increased the effective panicle number at maturity stage. Additionally, deeper root development significantly enhanced antioxidant enzyme activity in rice (Saini et al., 2018), reducing oxidative stress and maintaining normal metabolic function in seed cells (You et al., 2012). A more active antioxidant system extends the rice growth cycle (Zhang et al., 2015), leading to improved seed setting rates. In this study, compared to the broadcasting fertilizer application method, rice under the full root-zone fertilizer application method showed the highest mean value of SPAD, tiller number, SOD and POD activities, and superior root characteristics, further supporting the above explanation. This finding is consistent with the studies of (Xu et al., 2018). Also, The full root-zone fertilizer application method has similar performance to the deep side fertilizer application method, but overall it is better. The economic benefits directly reflect the direct impact of rice production on farmers (Pan et al., 2017). Net income can directly reflect the advantages and disadvantages of rice fertilization methods. Compared with traditional fertilization methods, RF method reduces manual labor and increases the total value of rice, thereby improving net income. This also meets the demand of farmers for improving rice productivity.

### 4.4 Effects of different fertilizer application methods on NUE

In traditional broadcast fertilizer application, dissolved fertilizers on the soil surface are prone to runoff losses with surface water (Hart et al., 2004), and the nitrogen elements entering the soil, such as nitrate nitrogen (NO_3_^^−^^), tend to leach into deeper soil layers and groundwater systems (Chen et al., 2021), especially under excessive rainfall or irrigation. Furthermore, High temperatures also can exacerbate nitrogen volatilization (Li et al., 2018). Both full root-zone and deep-side fertilizer application methods demonstrated higher nitrogen absorption (N_A_), nitrogen agronomic efficiency (N_AE_), and nitrogen recovery efficiency (N_RE_), compared to manually broadcasting method in this study, as the effectiveness of deep fertilizer applied method in mitigating nitrogen loss (Liu et al., 2015). By placing nitrogen fertilizers deeper in the soil near the root zone, both deep-side and full root-zone fertilizer application significantly reduce nitrogen loss at the soil surface (Li et al., 2022b). These methods also enhance direct nitrogen absorption by roots, minimizing nitrogen loss during soil infiltration (Aulakh, 1996). Moreover, the efficiency of nitrogen absorption by rice depends on the robustness of the root system (Mahboob et al., 2023). In this study, full root-zone and deep side fertilizer application both achieved the higher nitrogen absorption, NAE, and NRE than broadcast fertilizer application method. This is because both two methods effectively improved soil structure and enhanced root characteristics, enabling the roots to absorb more nitrogen. From the perspective of fertilizer spatial distribution, full root-zone fertilizer application promotes the direct absorption of nitrogen by roots, thereby improving nitrogen use efficiency, as the fertilizer is distributed more uniformly and closer to the root zone.

### 4.5 Implications, limitations, and prospects of the experiments

This study compared the effects of different fertilizer application methods on rice indicators and determined that RF method has significant agronomic advantages compared to traditional methods, and also has some advantages compared to DF method. Especially, the new method can improve the engineering problem of fertilizer blockage in the DF method on mechanical components, and also can effectively replace DF and traditional broadcasting method to improve rice productivity. However, these experiments also have some limitations. This study only focused on the application methods of basic fertilizers for rice, and traditional fertilizer application methods were used in the later stages. This study only analyzed the root characteristics of rice in the early stage of growth and did not track the root system of rice in the later stage. Future research should compare fertilization methods across the entire growth cycle. As this study was limited to conventional indica rice in South China, further testing across diverse soils, varieties and regions is recommended. Additional focus should be placed on how new methods affect soil properties, nitrogen loss, and agricultural sustainability.

## 5 Conclusion

A new nitrogen fertilizer application method, full root-zone fertilizer application, was introduced to rice production to address the disadvantages and limitations of conventional nitrogen fertilization methods widely used during rice transplanting. Results demonstrated that the full root-zone fertilizer application significantly improved early root development in rice plants by modifying the soil structure around the root zone. These improvements facilitated nitrogen uptake, increased tiller number, and enhanced the effective panicle number at maturity. The well-developed root systems also strengthened antioxidative enzyme activity, prolonged the growth cycle, and raised both grain number per panicle and seed-setting rate, collectively contributing to higher overall rice yield. Moreover, the full root-zone method reduced nitrogen loss during application and promoted root nitrogen absorption, leading to improved nitrogen use efficiency. In addition, this approach offered increased economic benefits by lowering labor input and elevating rice yield. This fertilizer application method achieves high rice yield and NUE, meeting farmers’ demands for improved productivity and economic benefits. Furthermore, it lays the groundwork for future research aimed at reducing nitrogen fertilizer application rates to support low-input rice production. However, the new method also has some limitations, and further exploration is needed to determine its universality for soil and rice types.

## Data Availability

The original contributions presented in the study are included in the article/**Supplementary Material**. Further inquiries can be directed to the corresponding author/s.
