# Supplementary figures and images for: Full root-zone nitrogen fertilizer application during rice transplanting promoted root development to increase rice yield and improve nitrogen use efficiency in South China
Source: Front Plant Sci. 2026 Jan 19;16:1715187. doi: 10.3389/fpls.2025.1715187 (PMC12861886; doi:10.3389/fpls.2025.1715187)

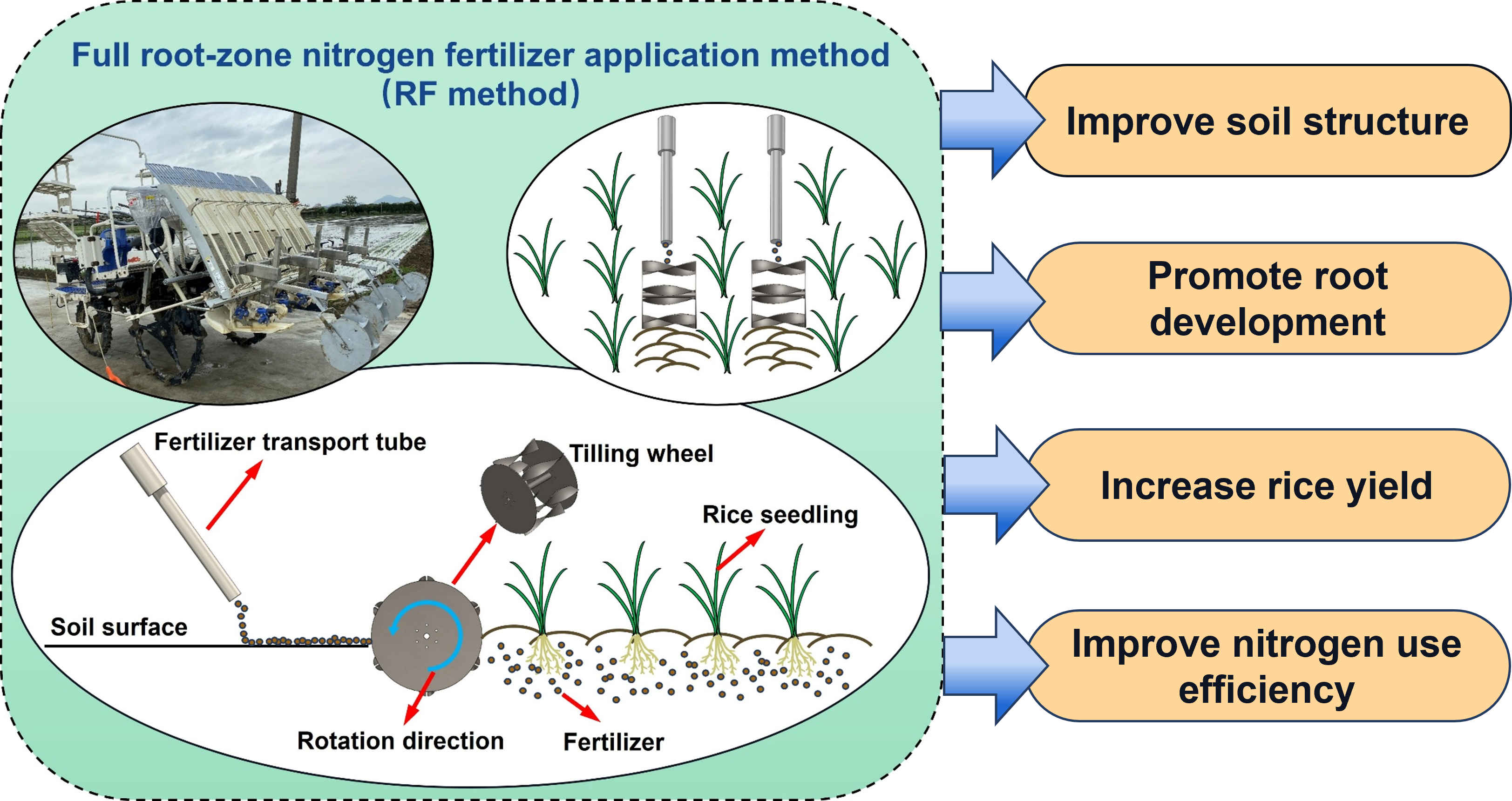

Supplement: Supplementary file 1 [file Image1.tif]
